# Reconciling Functional MRI Findings With Intraoperative Anatomy in Brain Tumor Surgery: Stereotactic-Guided Resection of Glioma in Broca’s Area

**DOI:** 10.7759/cureus.9220

**Published:** 2020-07-16

**Authors:** Constantin Fabian, Joe C Watson

**Affiliations:** 1 Neurosurgery, MedStar Georgetown University Hospital, Washington, DC, USA; 2 Neurology, Inova Fairfax Hospital, Falls Church, USA

**Keywords:** stereotactic navigation, surgery, brain tumors cns tumors, fmri, awake craniotomy, broca’s, oligodendroglioma

## Abstract

Stereotactic navigation and functional magnetic resonance imaging (fMRI) are increasingly used as important tools for intraoperative guidance and preoperative mapping for lesions in eloquent areas. We report a case in which a WHO grade II oligodendroglioma in Broca’s area with functional activation was successfully resected with the support of blood-oxygen-level-dependent imaging (BOLD)-fMRI mapping in a patient who refused an awake craniotomy. This case highlights key principles of tumor surgery navigation. Specifically, it calls into question the utility of awake craniotomy in this modern era. Ultimately, fMRI is an important tool for tumor resections and can limit the need for more expensive or invasive measures.

## Introduction

A key principal for brain tumor resection is to remove as much pathological tissue as possible while preserving functionally critical anatomical structures. Broca’s area, an essential structure for language function, raises a particular challenge due to variations in its location across individuals and its frequent involvement in frontotemporal tumors. Damage to this area can cause significant aphasia, which can be quite devastating to patients. As such, careful planning and decision making is of crucial importance in order to prevent loss in quality of life. 

In the “old days”, surgeons took advantage of the brain’s unique quality to not recognize pain within itself in order to permit awake operations and real-time assessment of function. Today, we have technology that allows us to identify functional brain areas before surgery and correlate them with intraoperative landmarks. Awake craniotomies may have their place even today, but they are less common and perhaps, we would argue, too primitive for modern use. Furthermore, patients are often unable or unwilling to participate in long awake craniotomies. To assist in operative planning, various mapping techniques are used to localize eloquent areas. Positron emission tomography (PET), magnetoencephalography, and functional magnetic resonance imaging (fMRI) are noninvasive mapping techniques. Of these techniques, fMRI has become the most widely useful because of its noninvasive nature, relatively high spatial resolution, and accessibility [1]. fMRI has also been shown to be quite precise in the localization of the eloquent cortex [2].

We report a case of a 37-year-old woman with a left frontal WHO grade II oligodendroglioma that showed some speech activation in what appeared to be the inferior posterior margin of the tumor on fMRI. Based on this, we offered an awake operation. She was unwilling to participate in an awake craniotomy and was successfully treated via asleep craniotomy for tumor resection with the assistance of fMRI.

## Case presentation

A 37-year-old right-handed woman presented to neurology with complaints of recent bilateral hand numbness and weakness. Additionally, she had experienced brief, infrequent episodes of aphasia for two years as well as recent trouble opening her left eye after waking. She denied any headaches or double vision. MRI showed a 3.1 x 3.2 cm nonenhancing mass in the left frontal region consistent with low-grade glioma. The patient was then referred to neurosurgery. 

**Figure 1:**
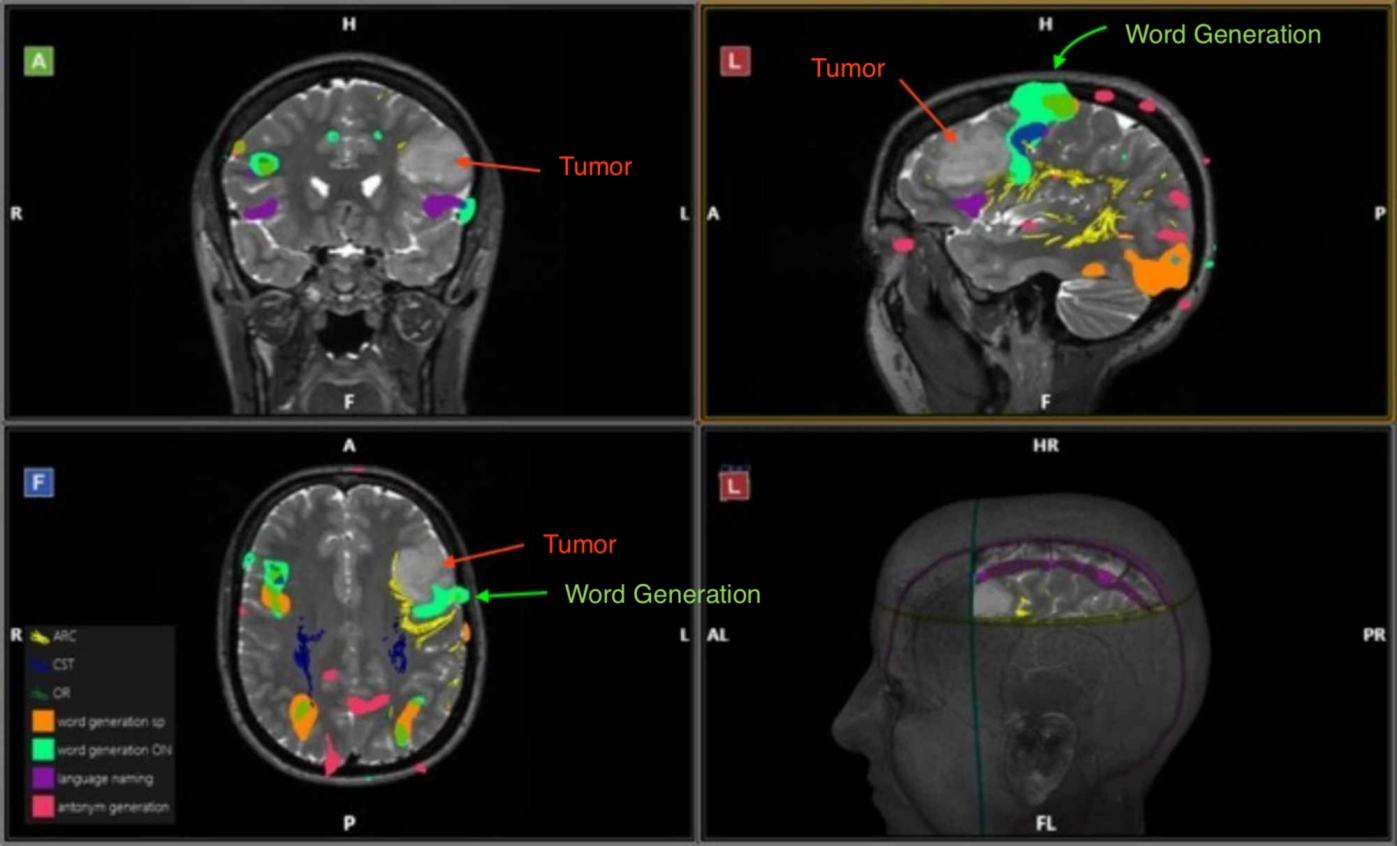
Functional MRI central view. Anterior, lateral, frontal, and lateral perspectives, respectively, show the tumor in the posterior inferior portion of the left frontal lobe, as well as activation for speech (word generation), highlighted in light green, near the inferior portion of the tumor. The tumor tissue is indicated by the red arrow, while the areas of functional activation are indicated by the green arrow.

Given the proximity of the lesion to the usual location of Broca’s area, an fMRI was ordered, along with our stereotactic protocol (Synaptive Medical, Toronto, Canada). A blood-oxygen-level-dependent (BOLD) fMRI was performed using picture naming, language tones naming tasks, antonym generation, American Society of Functional Neuroscience (ASFNR) sentence completion, and word generation. The structural images demonstrated a tumor in the posterior inferior portion of the left frontal lobe, involving the posterior portion of the left inferior frontal gyrus (Figure 1). The fMRI showed that the patient is left hemispheric dominant for speech. The fMRI further showed some activation on the tumor’s inferior-posterior and superior-posterior aspects with sentence completion, language tones naming, and word generation (Figure 1, light green). This corresponded to Broca’s area. 

We discussed the need for tissue diagnosis and concurrent resection with the patient. We offered an awake craniotomy due to the proximity of the lesion to Broca’s area; however, she firmly refused. Based on the fMRI, it seemed possible to remove the majority of the tumor while avoiding the eloquent cortex. As such, we decided to perform a left frontal craniotomy to obtain tissue diagnosis and to debulk the tumor. 

Under general anesthesia, using stereotactic computer-assisted guidance (Synaptive Medical, Toronto, Canada), we performed a left frontal craniotomy measuring approximately 4.5 x 4 cm. We opened the dura inferiorly and encountered abnormal brain. The middle and inferior frontal gyri were enlarged, with effacement of the sulcus in between. The frozen section biopsy confirmed a glioma. 

We marked the areas of eloquence from the preoperative MRI and correlated with the anatomy on the surface of the brain. This revealed that the tumor stopped directly anterior to Broca’s area. Therefore, it was not directly involved but simply exerted mass effect onto Broca’s area. 

We found the tumor to be slightly firmer and paler than the surrounding brain and therefore distinguishable. This distinction allowed us to use manual tactile dissection to separate the tumor from the healthy tissue. We dissected two significant arterial feeders away, which allowed us to then remove the tumor en bloc. We did not aggressively pursue margins for fear of causing injury to speech production or causing an infarct. 

**Figure 2:**
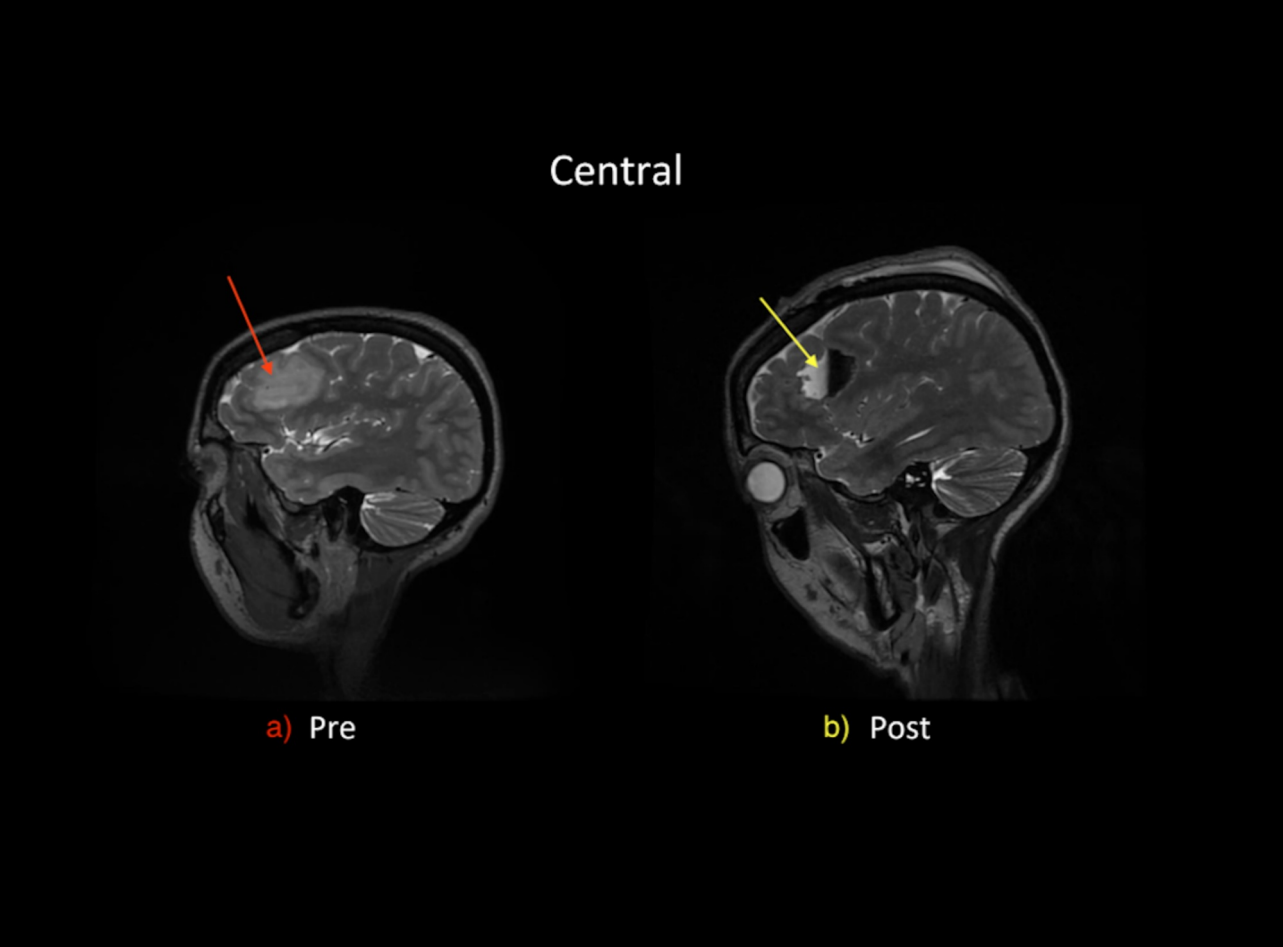
Central view of (a) preoperative MRI and (b) postoperative MRI. (a) The preoperative MRI shows tumor, 3.1 x 3.2 cm, in the posterior inferior portion of the left frontal lobe. (b) The postoperative MRI shows postoperative changes as fluid-filled resection cavity, 4.5 x 4 cm, in the posterior inferior portion of the left frontal lobe.

The patient’s postoperative course was uneventful. She had no complications or complaints and her speech returned to baseline. Final pathology revealed the tumor to be a WHO grade II oligodendroglioma with an IDH mutation and 1P/19Q co-deletion. The post resection MRI showed a resection cavity within the left frontal lobe containing fluid and blood products (Figure 2), as well as two areas of 12 x 7 mm of T2 and fluid-attenuated inversion recovery (FLAIR) hyperintense lesions near the posterior and anterior margins of the resection cavity, which reflect either residual tumor or postoperative edema. 

## Discussion

A fundamental goal before and during any surgery is to differentiate tumor tissue from functional tissue and any important structures, such as major vessels. This is especially important for any neurological regions that are highly eloquent such as Broca’s area, as any intervention carries the risk of significant impairments that can have far-reaching consequences in the patient's life. 

Traditionally, the precise functionality of the cortex can best be ascertained using invasive mapping techniques (IMT), such as intraoperative electrocortical stimulation (ECS). While quite reliable, ECS is highly invasive and significantly demanding for the patient. It is unavailable preoperatively. Since the birth of MRI, we have been awaiting the imaging sophistication to allow us to know these locations accurately before surgery, obviating the need for the awake craniotomy. Furthermore, other noninvasive techniquesm such as positron-emission tomography and magnetoencephalographym are used for surgical planning but are often expensive and unavailable, and not yet compatible with the most navigation software [3]. The fMRI is a noninvasive and widely available tool that can be used to gauge risk and help localization [4,5].

fMRI has been shown to be precise in functional area localization in the brain [2]. Intraoperatively, ECS remains the gold standard to some leaders in the field, but reports, such as this report, call that into question [6]. 

The expression “tumor don’t think” is a principal that is true of confluent tumor regions, especially in higher grade gliomas. Therefore, if the tumor presents to the cortical surface, dissection within the tumor mass in a subpial fashion is typically quite safe. Awake surgery is most useful when a tumor is more deeply seated and the surgeon must choose a pathway to the tumor that will cause the least amount of disruption of function. The “gray area” with respect to the indications for awake cranial surgery is for those tumors that are near eloquent areas such as Broca’s, Wernicke’s, or the primary motor cortex. With our new technology, these areas of eloquence are now able to be determined preoperatively, reducing significantly the indications for awake monitoring. 

In our case, the fMRI revealed that the tumor was close to Broca’s area, yet during surgery, correlation of the fMRI to the anatomy of the cortex revealed that the tumor stopped just in front of the precentral gyrus, marking an excellent point of separation. It showed that the tumor and the cortex were not aggressively entwined, thereby making the tumor safe for resection in that area. The case example demonstrates the limitation of fMRI with respect to spatial resolution for adjacent brain structures. Had we gone strictly by the fMRI data merged to the anatomical stereotactic MRI, we may have been reluctant to resect tumor in that region, which would have been a mistake. Therefore, we must recognize the limits of preoperative planning fMRI and stereotaxy. Surgeons will still need to rely on tactile and visual differences for safe tumor resection. 

It should be noted that awake craniotomies statistically show reduced resection and fewer postoperative deficits when compared to resections under general anesthesia [7,8]. However, as our case exemplifies an fMRI-guided resection is still safely possible, thereby providing a suitable alternative for patients unwilling to undergo an awake craniotomy. 

While this report only addresses fMRIs use in Broca’s area, the success can be extrapolated to other functional areas, for which precise localization is of key importance. 

## Conclusions

fMRI merged with anatomical stereotactic neuronavigation is a widely available tool to guide a resection that makes it both less invasive and a potential substitute for an awake craniotomy. This case exemplifies such an fMRI-supported resection and demonstrates some of its limitations.
